# Maternal Selenium and Iodine Nutritional Status and Perinatal Outcomes: A Scoping Review

**DOI:** 10.3390/nu18213521

**Published:** 2026-10-26

**Authors:** Hanchen Lin, Huanling Yu, Min Gao

**Affiliations:** 1 Beijing Key Laboratory of Environment and Aging, School of Public Health, Capital Medical University, Beijing 100069, China; 15011354809@163.com; 2 Department of Nutrition and Food Hygiene, School of Public Health, Capital Medical University, Beijing 100069, China

**Keywords:** selenium, iodine, pregnancy, maternal and infant outcomes, interaction effects, thyroid, placenta

## Abstract

**Objectives:** Selenium and iodine are essential trace elements whose requirements increase during pregnancy, jointly regulating thyroid function and placental homeostasis. While their individual effects have been extensively studied, the evidence on combined Se–I exposure and their underlying molecular interactions remains fragmented. This scoping review aimed to map the gestational dynamics of maternal selenium and iodine status, synthesize the evidence linking their independent and joint effects to perinatal outcomes, and summarize their potential interacting mechanisms. **Methods:** Following the PRISMA-ScR guidelines, PubMed, Web of Science, and Scopus were searched from 2016 to September 2026. Studies were eligible if they assessed maternal selenium and/or iodine status in pregnant women or mother–infant pairs and reported outcomes related to thyroid function, placental homeostasis, pregnancy complications, fetal development, or long-term offspring health. Data were charted using a standardized form and synthesized descriptively by nutrient exposure, biological axis, and outcome domain. **Results:** Of the 2198 records identified, 91 studies met the eligibility criteria (36 selenium-only, 53 iodine-only, and 2 combined Se–I). Evidence indicates that mild-to-moderate isolated or combined selenium and iodine deficiencies remain common in pregnancy and are associated with adverse perinatal outcomes. Mechanistic studies, largely from in vitro and animal models, support multi-level biological interactions between selenium and iodine in thyroid hormone activation, antioxidant pathways, and placental–maternal interface regulation. However, population-based evidence on combined exposure remains scarce, and no randomized controlled trial has evaluated the incremental clinical benefit of combined supplementation. **Conclusions:** Maternal selenium and iodine status are interrelated determinants of thyroid and placental function during pregnancy. Combined Se–I deficiency may be associated with a greater risk of adverse maternal and infant outcomes through overlapping and potentially interacting mechanisms; however, human evidence is insufficient to establish synergy. Future large-scale prospective cohorts and baseline-stratified randomized trials are urgently needed to establish an evidence base for status-guided integrated micronutrient screening and precision nutritional management during pregnancy.

## 1. Introduction

The first 1000 days of life are a critical window for the rapid development and functional maturation of fetal organ systems. Nutritional status during this period shapes lifelong health trajectories through developmental programming [1]. Globally, however, approximately 10–20% of pregnancies are affected by adverse outcomes, including spontaneous abortion, preeclampsia, preterm delivery, and fetal growth restriction [2], and maternal malnutrition is recognized as a principal modifiable risk factor [3,4]. Selenium (Se) and iodine (I) are essential trace elements whose physiological requirements increase substantially during gestation [5,6], leaving pregnant women particularly vulnerable to isolated or combined deficiencies of these micronutrients.

Selenium constitutes the active center of various selenoproteins, including glutathione peroxidases (GPX), thioredoxin reductases (TXNRD), and iodothyronine deiodinases (DIO), thereby playing critical roles in antioxidant defense, thyroid hormone (TH) metabolism, and immune regulation [7]. Iodine, in turn, primarily exerts its physiological effects through TH synthesis [8,9]. The functions of the two trace elements are, however, closely intertwined rather than independent: iodine is the obligatory substrate for TH synthesis, whereas selenium, in the form of selenocysteine (Sec), occupies the catalytic centers of the three deiodinase isozymes (DIO1, DIO2, and DIO3) that convert thyroxine (T4) to the more bioactive triiodothyronine (T3) [10]. Beyond the TH axis, the selenoproteins GPX and TrxR scavenge lipid peroxides [11,12], while iodide ions directly neutralize reactive oxygen species (ROS) [13], so the two elements act complementarily against oxidative stress. Together, these molecular interactions indicate that Se and I may act jointly and interact in TH homeostasis and redox regulation, an interplay that may jointly modulate pregnancy outcomes [14].

No existing review, however, has systematically integrated the physiological dynamics, combined exposure effects, and intervention outcomes of maternal selenium and iodine status during pregnancy, nor has it elucidated their underlying molecular synergistic mechanisms. This scoping review maps the gestational trajectories of the two micronutrients, synthesizes the epidemiological evidence linking their status to maternal and infant outcomes, and summarizes the interactive effects of combined supplementation on thyroid and placental function. In doing so, it offers a starting point for future research toward integrated micronutrient screening and precision nutritional management during pregnancy.

## 2. Materials and Methods

This review is reported in accordance with the Preferred Reporting Items for Systematic Reviews and Meta-Analyses extension for Scoping Reviews (PRISMA-ScR) (Table S1) [15].

### 2.1. Research Questions

- What is the association between selenium and/or iodine status and maternal thyroid function during pregnancy?
- What is the association between selenium and/or iodine status and pregnancy complications, including hypertensive disorders of pregnancy, gestational diabetes mellitus, preterm birth, miscarriage, and fetal growth restriction?
- What is the association between selenium and/or iodine status and offspring health and neurodevelopmental outcomes?

### 2.2. Literature Search Strategy

This scoping review followed the methodological framework of Arksey and O’Malley, as further refined by Levac et al. [15], and the Joanna Briggs Institute (JBI) methodology for scoping reviews, comprising five sequential stages: research question formulation, literature identification, study selection, data charting, and collating, summarizing, and reporting of the results. We systematically searched PubMed, Web of Science, and Scopus from 2016 to September 2026. The search combined terms related to selenium, iodine, pregnancy, thyroid function, placental function, and maternal and infant outcomes.

The full search strategy for PubMed is provided below as an example; the complete strategies for all databases are available from the corresponding author on request and are provided in the Supplementary Materials.

(“selenium”[ti] OR “iodine”[ti] OR “selenium”[mh] OR “iodine”[mh]) AND (((“pregnancy”[ti] OR “pregnant”[ti] OR “gestation”[ti] OR “pregnancy”[mh] OR “pregnant women”[mh]) OR (((“thyroid”[ti] OR “thyroid hormone”[ti] OR “thyroid hormones”[ti] OR “thyroid”[mh] OR “thyroid hormones”[mh]) AND (“pregnancy”[ti] OR “pregnant”[ti] OR “gestation”[ti] OR “pregnancy”[mh] OR “pregnant women”[mh])) OR (“placenta”[ti] OR “trophoblast”[ti] OR “placenta”[mh] OR “trophoblasts”[mh]) OR (“preeclampsia”[ti] OR “pre-eclampsia”[ti] OR “hypertensive disorders of pregnancy”[ti] OR “pregnancy-induced hypertension”[ti] OR “pre-eclampsia”[mh] OR “hypertension, pregnancy-induced”[mh]) OR (“gestational diabetes”[ti] OR “GDM”[ti] OR “gestational diabetes mellitus”[ti] OR “diabetes, gestational”[mh]) OR (“preterm birth”[ti] OR “premature birth”[ti] OR “spontaneous abortion”[ti] OR “miscarriage”[ti] OR “recurrent miscarriage”[ti] OR “premature birth”[mh] OR “abortion, spontaneous”[mh] OR “abortion, habitual”[mh]) OR (“fetal growth”[ti] OR “fetal growth restriction”[ti] OR “IUGR”[ti] OR “small for gestational age”[ti] OR “SGA”[ti] OR “birth weight”[ti] OR “fetal growth retardation”[mh] OR “infant, small for gestational age”[mh] OR “birth weight”[mh]) OR (“neurodevelopment”[ti] OR “cognitive”[ti] OR “IQ”[ti] OR “ADHD”[ti] OR “autism”[ti] OR “ASD”[ti] OR “Bayley”[ti] OR “WPPSI”[ti] OR “neurodevelopmental disorders”[mh] OR “cognition”[mh] OR “autism spectrum disorder”[mh] OR “attention deficit disorder with hyperactivity”[mh])) AND humans[mh] NOT (review[pt] OR systematic review[pt] OR meta-analysis[pt] OR scoping review[pt] OR editorial[pt] OR comment[pt] OR letter[pt] OR case reports[pt])

The search strategy was developed by the review team and refined through preliminary searches. The reference lists of included articles and relevant reviews were also checked for additional eligible studies. No restrictions were applied to study design or country of origin.

### 2.3. Study Selection and Eligibility Criteria

Records retrieved from PubMed, Web of Science, and Scopus were imported into reference management software, and duplicates were removed. Two reviewers (LH and GM) independently screened titles and abstracts, followed by full-text assessment of potentially eligible reports. Disagreements were resolved by discussion or by a third reviewer. Before formal screening, the eligibility criteria were pilot-tested on a random sample of records by both reviewers to ensure consistent application.

### 2.4. Inclusion Criteria

Studies were included if they (1) involved pregnant women, including postpartum and offspring follow-up cohorts; (2) assessed biomarkers of selenium or iodine status, such as serum, plasma, or whole blood selenium, selenoproteins, or urinary iodine; and (3) evaluated outcomes related to thyroid function, pregnancy complications, or offspring health.

### 2.5. Exclusion Criteria

Studies were excluded if they (1) were not published in English; (2) were not original research articles (i.e., reviews, editorials, comments, letters, conference abstracts, and case reports); or (3) did not report outcomes related to thyroid function, pregnancy complications, or offspring health. Studies for which the full text could not be obtained were also excluded.

**Figure 1:**
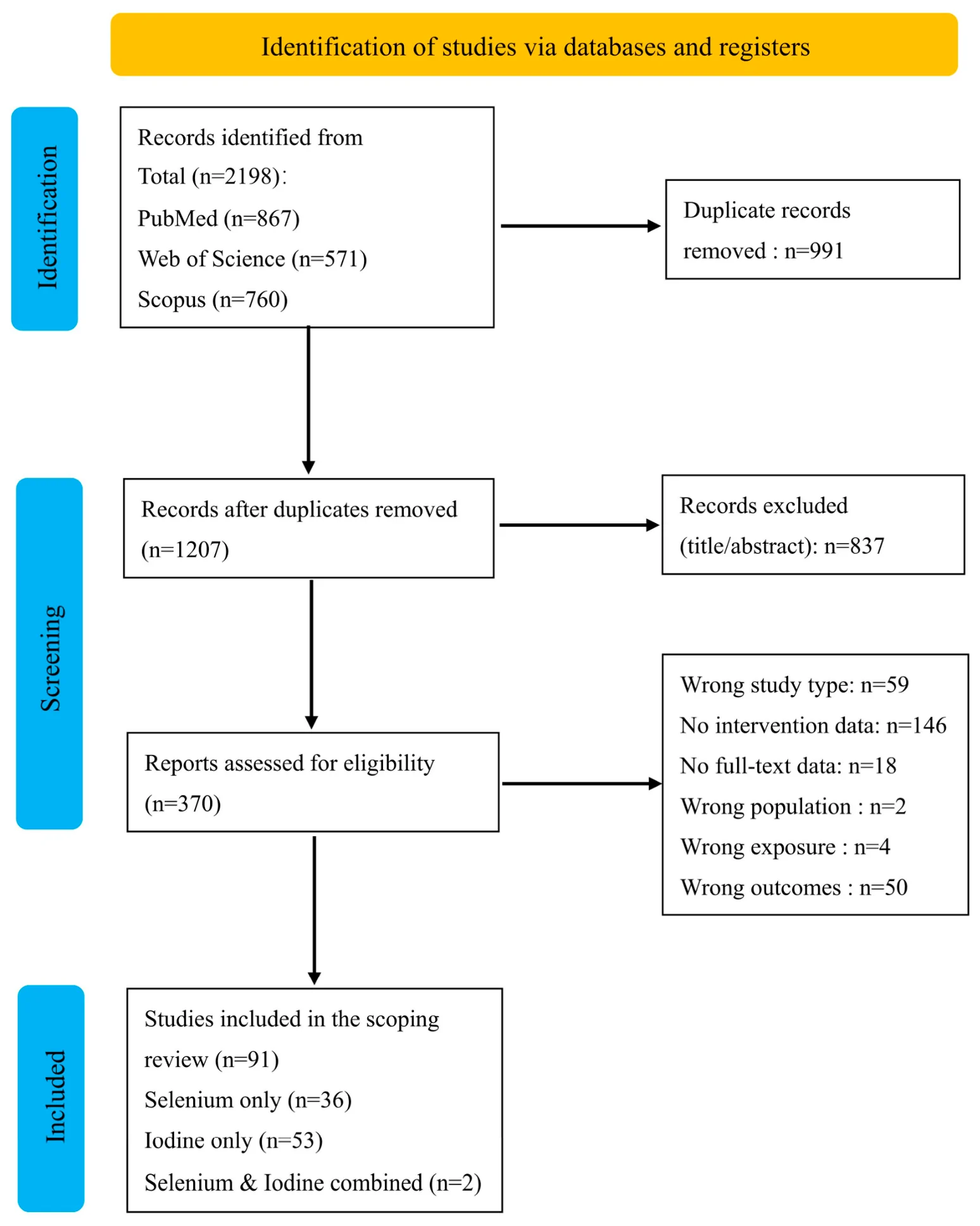
PRISMA-ScR flow diagram of study selection.

As shown in Figure 1, a total of 2198 records were identified: PubMed (*n* = 867), Web of Science (*n* = 571), and Scopus (*n* = 760). After removing 991 duplicates, 1207 records remained. Title and abstract screening excluded 837 records, leaving 370 reports for full-text assessment. Of these, 279 reports were excluded for the following reasons: wrong study type (*n* = 59), no relevant data (*n* = 146), full text unavailable (*n* = 18), wrong population (*n* = 2), wrong exposure (*n* = 4), and wrong outcomes (*n* = 50). Finally, 91 studies were included in the scoping review: selenium only (*n* = 36), iodine only (*n* = 53), and combined selenium and iodine (*n* = 2). The selection process is summarized in Figure 1.

### 2.6. Data Extraction

Data charting was performed independently by two reviewers (LH and GM) using a standardized extraction form that was pilot-tested on five included studies and refined before use. Disagreements were resolved by discussion or by a third reviewer. The following information was extracted from each included study: first author and year of publication, country, study design, sample size and participant characteristics, trimester and method of selenium or iodine biomarker assessment, thyroid function measures, pregnancy and offspring outcomes, statistical methods, and key findings.

### 2.7. Data Summarized

Extracted data were synthesized qualitatively using a narrative approach. Studies were grouped thematically according to the three research questions, and the findings were summarized in structured evidence tables and in the text. Consistent with the JBI methodology for scoping reviews, a formal critical appraisal of the methodological quality or risk of bias of included studies was not performed.

## 3. Results

### 3.1. Association of Maternal Selenium Nutritional Status with Maternal and Infant Outcomes

#### 3.1.1. Selenium Intake, Nutritional Assessment, and Status During Pregnancy

Epidemiological evidence indicates that selenium deficiency during pregnancy is a global concern. A survey of 366 pregnant women in Shunyi District, Beijing, China, reported a selenium deficiency prevalence, defined as serum selenium < 70 μg/L, as high as 66.9% during early gestation [16]. Similarly, in a Polish observational study conducted in a region recognized as selenium-deficient, Filipowicz et al. [17] found that 79% of pregnant women had serum selenium below 70 μg/L and that 22% fell into the severely deficient category (<45.9 μg/L). The same study demonstrated strong positive correlations between maternal serum selenium, SELENOP concentrations, and GPX3 activity and the corresponding neonatal cord blood indicators (all *p* < 0.001), suggesting effective transplacental transfer of maternal selenium status to the fetus. Low-dose selenium supplementation (mean dose 42 ± 14 μg/d), however, proved insufficient to correct deficiency in either mothers or newborns; only when the daily dose exceeded 55 μg did maternal SELENOP concentrations measurably increase.

Dietary reference intake recommendations differ modestly among national guidelines [18]. The Australian National Health and Medical Research Council (NHMRC) recommends 65 μg/day of selenium for pregnant women, the U.S. National Institutes of Health (NIH) recommends 60 μg/day, and China’s Dietary Reference Intakes for Chinese Residents (2023 Edition) recommends 65 μg/day during pregnancy.

#### 3.1.2. Selenium and Maternal Thyroid Function and Pregnancy Complications

Several studies have investigated the association between maternal selenium status and thyroid autoimmunity during pregnancy. In a prospective study within the Korean Ideal Breast Milk Cohort (367 women in mid-to-late pregnancy), Chung et al. [19] reported a thyroid peroxidase antibody (TPOAb) positivity rate of 13.3% in the plasma selenium deficiency group (<70 μg/L), significantly higher than the 4.6% observed in the sufficient group (≥100 μg/L; *p* = 0.031). The proportion of thyroid ultrasound parenchymal heterogeneity was 33.3% in the deficiency group, markedly higher than in the suboptimal (14.6%) and sufficient (17.3%) groups (*p* = 0.042). In the Italian SERENA study [20], intervention with 83 μg/d L-selenomethionine in pregnant women with thyroiditis led to significantly lower thyroglobulin antibody (TgAb) (19.86 vs. 151.03 IU/L, *p* < 0.01) and TPOAb (255.00 vs. 441.28 IU/L, *p* < 0.01) levels at 6 months postpartum than placebo.

The geometric mean serum soluble fms-like tyrosine kinase 1 (sFlt1) at 35 weeks was approximately 30% lower than in the placebo group (5.22 vs. 7.49 ng/mL, *p* = 0.039). In high-risk pregnant women, a higher dose (200 μg/d from 16–18 weeks for 12 weeks) significantly reduced the uterine artery pulsatility index (β = −0.09, *p* = 0.04) and improved oxidative and inflammatory biomarkers, including total antioxidant capacity (TAC), glutathione (GSH), and high-sensitivity C-reactive protein (hsCRP) [21].

A prospective cohort study by Lewandowska et al. [22] (*n* = 484) found that lower first-trimester (10–14 weeks) serum selenium levels were associated with a higher subsequent risk of HDP. The mean serum selenium level in the HDP group was 57.51 μg/L, significantly lower than 62.89 μg/L in the control group (*p* < 0.001). In never-smokers with a normal pre-pregnancy body mass index (BMI), the adjusted odds ratio (AOR) for HDP in the lowest quartile (≤57.68 μg/L), compared with the highest quartile (>66.60 μg/L), was 15.34 (95% CI: 2.73–86.31, *p* = 0.002).

A nested case–control study involving 678 pregnant women [23] showed that, compared with the highest tertile of urinary selenium (>26.77 μg/g creatinine), the lowest tertile (<18.10 μg/g creatinine) carried a 2.35-fold higher risk of gestational diabetes mellitus (GDM) (OR = 2.35, 95% CI: 1.36–4.06, *p* = 0.01). A further prospective cohort study (*n* = 398) [24], however, found no causal association between first-trimester selenium exposure and GDM (OR = 1.511, 95% CI: 0.815–2.803).

#### 3.1.3. Selenium and Placental Function and Pregnancy Outcomes

Beyond its associations with maternal pregnancy complications, selenium deficiency has been implicated in placental dysfunction and adverse perinatal outcomes. A prospective cohort study of 2519 pregnant women [25] demonstrated that maternal selenium levels in the second trimester were significantly inversely correlated with placental mRNA expression of stress- and inflammation-related markers, including HO-1, HIF1α, and GRP78 (β = −0.011 to −0.005 for each marker, all *p* < 0.05). An epigenetic study [26] found that for each IQR increase in placental selenium levels, the risk of neonatal hypotonia increased 1.76-fold (OR = 1.76, 95% CI: 1.12–2.82).

An RCT among HIV-infected pregnant women in Nigeria [27] found that daily supplementation with 200 μg selenium significantly reduced the risk of preterm birth (PTB) (5.6% in supplementation group vs. 15.6% in placebo group, RR = 0.32, 95% CI: 0.11–0.96), with further risk reduction for those using it for more than 14 weeks (RR = 0.32, 95% CI: 0.11–0.86). However, a secondary analysis study based on women from Guatemala, India, and Pakistan [28] did not find a significant association between selenium concentration at 12 or 34 weeks of gestation and PTB (OR = 1.004, 95% CI: 0.989–1.084, *p* = 0.568).

A case–control study of patients with recurrent miscarriage (RM) in Saudi Arabia [29] showed that plasma selenium levels were significantly lower in patients with RM (75.3 ± 11.2 μg/L) than in healthy pregnant women (87.6 ± 12.6 μg/L) and non-pregnant controls (96.3 ± 13.5 μg/L), with similar decreasing trends in whole blood and placental tissue selenium (all *p* < 0.001). A further study [30] found that plasma selenium in patients with RM was reduced by about 18% (0.78 ± 0.09 vs. 0.95 ± 0.13 μmol/L) and placental tissue selenium by about 29% (12.0 ± 1.73 vs. 16.8 ± 2.26 nmol/g, *p* < 0.0001), relative to healthy pregnant women. Expression of the antioxidant enzyme genes GPX, glutathione reductase (GSR), superoxide dismutase (SOD), and catalase (CAT) was significantly downregulated in placental tissue (fold changes 0.65, 0.46, 0.61, and 0.55, respectively; *p* < 0.001).

A Polish nested case–control study (*n* = 240) [31] showed that pregnant women with first-trimester serum selenium concentrations at or below 56.6 μg/L had an approximately three-fold higher risk of delivering a small-for-gestational-age (SGA) infant (OR = 3.02, *p* = 0.019).

In the Odense Child Cohort in Denmark (*n* = 719) [32], each 10 μg/L increase in third-trimester maternal serum selenium was associated with a 24% lower risk of attention deficit hyperactivity disorder (ADHD) traits (OR = 0.76, 95% CI: 0.60–0.94) and a 15% lower risk of autism spectrum disorder (ASD) traits (OR = 0.85, 95% CI: 0.74–0.98) in offspring at 5 years of age. A Polish prospective cohort (*n* = 410) [33] found that first-trimester maternal plasma selenium levels correlated positively with motor development at 1 year (β = 0.2, *p* = 0.002) and language development at 2 years (β = 0.2, *p* = 0.03) in offspring. The Boston Birth Cohort in the USA (*n* = 1550) [34], however, observed the opposite pattern: each IQR (72 μg/L) increase in maternal erythrocyte selenium was associated with a 49% higher risk of ASD (OR = 1.49, 95% CI: 1.09–2.02) and a 29% higher risk of ADHD (OR = 1.29, 95% CI: 1.04–1.56) in offspring. Table 1 and Table 2 summarize studies related to maternal selenium status and maternal and infant outcomes.

### 3.2. Association of Maternal Iodine Nutritional Status with Maternal and Infant Outcomes

#### 3.2.1. Iodine Intake, Nutritional Assessment, and Status During Pregnancy

Iodine is an essential trace element and a critical substrate for TH synthesis. During pregnancy, increased TH production, an elevated glomerular filtration rate, and fetal thyroid development together drive an approximately 50% increase in iodine requirements relative to the non-pregnant state [8,9].

Urinary iodine concentration (UIC) is currently the primary biomarker recommended by the World Health Organization (WHO) for assessing population iodine status. Because UIC reflects short-term iodine intake (hours to days) and varies substantially from day to day within individuals, depending on hydration status and urine output, the WHO advises against using single measurements for individual assessment. Instead, the population median UIC, derived from at least 100–500 samples, is recommended for evaluating population-level iodine nutrition. According to WHO criteria, the iodine status of pregnant populations is categorized as insufficient (median UIC < 150 μg/L), adequate (150–249 μg/L), above requirements (250–499 μg/L), or excessive (≥ 500 μg/L) [35].

Globally, iodine deficiency persists as a significant public health concern. A disease burden analysis encompassing 204 countries [36] estimated the age-standardized disability-adjusted life year (DALY) rate for iodine deficiency at 27.66 per 100,000 population in 2021. Even in China, where universal salt iodization has been implemented for decades, the iodine status of pregnant women has fluctuated in recent years: a continuous monitoring study by Wu et al. [37] in Beijing from 2021 to 2024 reported a decline in median UIC from 151.3 μg/L to 122.9 μg/L, with deficiency particularly pronounced during the first trimester.

Recommendations regarding iodine supplementation during pregnancy vary slightly across international authorities. Both the American Thyroid Association (ATA) and the Endocrine Society recommend a daily supplement containing 150 μg of iodine for pregnant women [38,39]. The WHO considers that pregnancy increases iodine requirements by approximately 100 μg/day above non-pregnant levels [35], whereas the European Food Safety Authority (EFSA) suggests a more modest increment of about 50 μg/day [40].

#### 3.2.2. Iodine and Maternal Thyroid Function and Pregnancy Complications

A randomized double-blind trial in Sweden (*n* = 200) [41] showed that daily supplementation with 150 μg iodine increased maternal UIC from a baseline of 110 μg/L to 139 μg/L in the second trimester and 136 μg/L in the third. Serum thyroglobulin (Tg) levels in the supplementation group were significantly lower than in the control group in both trimesters (16 vs. 20 μg/L, *p* = 0.010; 22 vs. 31 μg/L, *p* = 0.003), with no significant differences in thyroid-stimulating hormone (TSH), free thyroxine (FT4), or TPOAb positivity rates. González-Martínez et al. [42] found that among pregnant women without autoimmune thyroid disease, iodized salt users had significantly lower TSH than non-users (2.08 ± 0.89 vs. 2.56 ± 1.02 mIU/L, *p* = 0.025), whereas among those with pre-existing autoimmune thyroid disease, iodine supplement users had paradoxically higher TSH (2.97 ± 1.25 vs. 1.16 ± 0.41 mIU/L, *p* = 0.002). A nationwide cross-sectional study in the Faroe Islands (*n* = 623) [43] found that serum Tg increased significantly only in the UIC < 50 μg/L group (*p* = 0.02), while TSH increased with rising UIC (*p* < 0.001).

A case–control study [44] showed that UIC was significantly lower in the preeclampsia group than in the control group (90.0 vs. 351.0 μg/L, *p* < 0.0001), and a UIC threshold of <239 μg/L predicted preeclampsia with an area under the curve (AUC) of 0.920 (95% CI: 0.893–0.946, *p* < 0.0001). In contrast, in the large Norwegian Mother, Father and Child Cohort Study (MoBa, *n* = 78,318) [45], dietary iodine below 100 μg/d among non-supplemented women was associated with an increased risk of preeclampsia (75 vs. 100 μg/d, aOR = 1.14, 95% CI: 1.08–1.22, *p* < 0.001), whereas long-term pre-pregnancy iodine supplementation reduced the risk (aOR = 0.85, 95% CI: 0.74–0.98, *p* = 0.022).

In another Norwegian MoBa cohort (58,629 pregnant women) [46], iodine intake below 100 μg/d was associated with a 29% higher risk of pharmacologically treated hypertension within 10 years postpartum, relative to intake of 150–199 μg/d (aHR = 1.29, 95% CI: 1.09–1.53). Within the HDP subgroup, the risk increased to 35% (aHR = 1.35, 95% CI: 1.00–1.83). Even after excluding women with pregnancy complications, low iodine intake remained independently associated with long-term hypertension (aHR = 1.46). A cross-sectional study of 250 pregnant women with HDP [47] showed that the median UIC was only 82.5 μg/L and that 76.4% exhibited iodine deficiency (UIC < 150 μg/L). The median UIC of patients with preeclampsia (61.3 μg/L) was significantly lower than that of women with gestational hypertension (111.6 μg/L, *p* = 0.018), and systolic blood pressure was negatively correlated with UIC (β = −0.006, *p* = 0.015); this association was not accompanied by changes in TSH or anti-thyroid peroxidase (anti-TPO) antibodies.

In iodine-deficient areas, the median UIC in women with GDM was significantly lower than in healthy controls (89.50 vs. 150.05 μg/L, *p* < 0.001), and the proportion with iodine deficiency reached 88.7% in the GDM group versus only 50% in the control group [48]. Conversely, in an iodine-sufficient area, a first-trimester serum iodine concentration (SIC) above the median (88.9 μg/L) was significantly positively correlated with blood glucose (β = 0.062, *p* < 0.001) [49].

#### 3.2.3. Iodine and Placental Function and Maternal and Infant Outcomes

Peng et al. [50] found that maternal-side placental iodine content (1.38 ± 0.32 μg/g) was significantly higher than fetal-side content (0.45 ± 0.13 μg/g, *p* < 0.01), and maternal-side iodine content correlated positively with UIC (r = 0.316, *p* < 0.05). Fu et al. [51] found that placental iodine storage (PIS) correlated positively with both UIC (R = 0.58, *p* < 0.001) and serum iodine concentration (R = 0.55, *p* < 0.001), and that PIS was significantly higher in the excessive iodine intake group than in the adequate group (*p* = 0.017).

An Iranian prospective cohort [8] showed that among pregnant women with UIC <100 μg/L and TSH ≥ 4 μIU/mL, the risk of preterm birth increased 2.5-fold (OR = 2.5, 95% CI: 1.1–10.0, *p* = 0.024). The preterm birth rate in the UIC <100 μg/L group was significantly higher than in the UIC ≥ 250 μg/L group (9% vs. 4%, *p* = 0.015).

A Portuguese retrospective study [52] showed that after the 2013 national recommendation for iodine supplementation during pregnancy (150–200 μg/d), the supplementation rate increased from 25% to 81%. Iodine supplementation was significantly negatively correlated with the number of adverse pregnancy outcomes such as miscarriage (OR = 0.791, 95% CI: 0.651–0.960, *p* = 0.018). A cross-sectional study in the plateau area of Qujing, China [53], reported median UIC values of 123, 136, and 123 μg/L in the first, second, and third trimesters, respectively, with an overall iodine deficiency rate (UIC < 150 μg/L) of 59.4%. Multivariable logistic regression found no statistically significant association between iodine deficiency and miscarriage risk (OR = 0.69, 95% CI: 0.24–1.97, *p* = 0.487). Although the miscarriage rate was higher in the more-than-adequate or excessive iodine group (9.38% vs. 5.22%), the difference was not significant (OR = 1.99, 95% CI: 0.67–5.91, *p* = 0.217).

The Hangzhou cohort in China [54] showed that lower third-trimester maternal UIC was associated with lower neonatal birth weight and length, while lower first-trimester UIC was associated with reduced offspring weight and length at 18 months of age. In the Norwegian MoBa cohort (*n* = 78,318) [45], iodine intake of 75 μg/day, relative to 150 μg/day, was associated with a 0.08 lower birth weight z-score (approximately 36 g). Using the iodine-to-creatinine ratio (I:Cr) to correct for urine dilution, the UK BiB cohort [55] predicted a 41 g higher birth weight and a 1.9 percentage point lower probability of SGA when I:Cr increased from 59 to 121 μg/g. The Wuhan cohort [56] found that first-trimester iodine deficiency (I:Cr < 150 μg/g) was significantly associated with slower fetal femur length growth in the second and third trimesters.

An RCT conducted in Thailand and India (*n* = 832) [57] showed that daily supplementation with 200 μg iodine from the first trimester until delivery had no significant impact on verbal IQ, performance IQ, or executive function scores on the Wechsler Preschool and Primary Scale of Intelligence (WPPSI-III) in offspring at 5–6 years of age. Moreover, in a study of pregnant women with subclinical hypothyroidism or hypothyroxinemia [58], offspring IQ at 5 years of age did not differ significantly between the group with UIC < 150 μg/L and the iodine-sufficient group. Table 3 and Table 4 summarize studies related to maternal iodine status and maternal and infant outcomes.

#### 3.2.4. Maternal and Infant Health Effects of Combined Selenium and Iodine Exposure

Population-based studies that simultaneously assess both selenium and iodine nutritional status during pregnancy remain scarce, and none to date have reported composite maternal or infant clinical outcomes in relation to dual exposure. In a cross-sectional survey of pregnant Dutch women, Mayunga et al. [59] described the concurrent inadequacy of dietary selenium and iodine intake: 61% of pregnant women had insufficient intake of both trace elements in the same individual. Importantly, however, this study only reported the prevalence of combined deficiency and did not extend to analyzing its association with any maternal or perinatal endpoints.

#### 3.2.5. Selenium and Iodine in Thyroid Metabolism

Gustin et al. [14] studied Swedish pregnant women with mild selenium deficiency (median plasma selenium 67 μg/L) combined with iodine deficiency (median UIC 112 μg/L). Plasma selenium concentration was significantly negatively correlated with free T3 (fT3) and total T3 (tT3) levels (fT3: β = −0.005, *p* = 0.001; tT3: β = −0.006, *p* = 0.001). The study, however, did not report associations of combined exposure with clinical outcomes such as preeclampsia, GDM, or fetal growth, and no other population studies to date have directly evaluated the impact of combined selenium and iodine exposure on maternal thyroid function or gestational thyroid disease. In FRTL-5 rat thyroid cells, Oglio et al. [60] found that selenium supplementation significantly enhanced iodide-induced Nrf2 transcriptional activity (luciferase reporter assay), an effect that iodine or selenium supplementation alone did not achieve to the same degree. Contempre et al. [61,62] observed that in selenium-deficient rats, a high-iodine diet induced severe TGF-β-mediated thyroid fibrosis, whereas adequate selenium pre-supplementation prevented this damage. Foroughi et al. [63] found in primary cultured fetal thyroid cells from sheep and cattle that sodium selenite supplementation significantly upregulated the transcription of DIO1 and DIO2.

#### 3.2.6. Selenium and Iodine in Placental Function Regulation

Kurlak et al. [64] observed a strong positive correlation (r > 0.5, *p* < 0.05) between DIO3 mRNA levels and enzymatic activity in placental tissue from patients with preeclampsia, an association that appeared only in the pathological state. The study discussed the potential association between selenium nutritional status and iodine metabolic enzyme activity but did not directly measure maternal selenium levels. No population studies to date have directly assessed the association of combined selenium and iodine exposure during pregnancy with placental development or functional indicators such as placental weight, blood flow resistance, or transport efficiency.

Habibi et al. [65] found that in HTR-8/SVneo trophoblast cells under oxidative stress, supplementation with 2.8 μM sodium selenite alone reduced lipid peroxidation to 70% of control levels, whereas combined supplementation with 2.8 μM sodium selenite and 1 μM potassium iodide reduced it to 90% of control levels; the combined effect was significantly superior to selenium alone (*p* < 0.05). In first-trimester placental explants, combined supplementation (0.8 μM sodium selenite plus 0.5 μM potassium iodide) also alleviated trophoblast apoptosis and oxidative DNA damage more effectively than either supplement alone [66]. Olivo-Vidal et al. [67] found in BeWo trophoblast cells that iodine deficiency increased ROS levels by 47.5% within 24 h (*p* < 0.05), accompanied by upregulation of PPAR-γ, Snail, and MMP-9 mRNA and downregulation of GCM-1, hCG, and E-cadherin mRNA. Low-dose iodine (5–500 μM) reversed these changes, whereas high-dose iodine (1000–5000 μM) exhibited cytotoxicity.

Li et al. [68] established a selenium-deficient mouse model of pregnancy (0.02 vs. 0.2 mg/kg Se in controls). The selenium-deficient group showed significantly increased placental H_2_O_2_ content, decreased catalase and glutathione levels, suppressed proliferating cell nuclear antigen (PCNA) expression, increased autophagy-related proteins (LC3B, Beclin1) and pro-apoptotic proteins (Bax, cleaved caspase-9/-3), and decreased anti-apoptotic Bcl2. Subsequent transcriptomic analysis by the same team [69] found that selenium deficiency significantly inhibited glutathione peroxidase activity, increased malondialdehyde content, reduced serum progesterone levels by downregulating Hsd3b1 expression, decreased the expression of various ion transporters, and increased placental macrophage numbers and the expression of the pro-inflammatory chemokines Ccl3, Ccl8, Cxcl10, and Cxcl14. Hofstee et al. [70] found in a selenium-deficient mouse model of pregnancy (Se < 50 μg/kg) that maternal and fetal T3 and T4 concentrations were significantly higher than in selenium-adequate controls, while placental DIO2 and DIO3 mRNA expression decreased (*p* < 0.05), accompanied by fetal growth restriction (reduced birth weight).

## 4. Discussion

This scoping review provides a systematic synthesis of the available evidence on the impact of maternal selenium and iodine nutritional status on maternal and infant health. Distinct from prior reviews that focused predominantly on single nutrients, the present work delineates the independent roles of selenium and iodine in maternal physiology and synthesizes their potential molecular interaction mechanisms across three interconnected domains: thyroid hormone metabolism, antioxidant and anti-inflammatory pathways, and placental functional regulation. Our synthesis reveals that the effects of selenium exhibit a clear functional threshold dependence, whereas those of iodine follow a U-shaped dose–response relationship. Despite mechanistic evidence supporting multilevel interactions between selenium and iodine, population-based data on combined exposure and its health implications remain conspicuously absent. This represents a critical knowledge gap that warrants prioritized investigation in future maternal nutrition research.

When blood selenium exceeds approximately 95–110 μg/L, the enzymatic activity of selenoproteins such as GPX and TrxR no longer increases with additional supplementation, indicating that the regulatory effect of selenium saturates above this concentration. A 2024 dose–response meta-analysis of 18 population cohorts [71] showed that antioxidant enzyme activity increased linearly with selenium levels when blood selenium was <100 μg/L. Beyond the 100 μg/L threshold, GPX and TrxR showed no significant gains, and high selenium exposure instead increased the risk of metabolic abnormalities. A meta-analysis of RCTs of selenium supplementation for Hashimoto’s thyroiditis [72] showed that only individuals with baseline blood selenium below 105 μg/L benefited, in terms of improved selenoprotein activity and reduced thyroid autoantibodies; those with blood selenium in the saturation range showed no significant effects. The saturation threshold in the pregnant women subgroup was stable at 98–110 μg/L. In UK primiparous women with baseline whole blood selenium in the lowest quartile, supplementation reduced preeclampsia risk by 65% [73], whereas RCTs in parts of China [16] failed to show significant benefits. These discrepancies suggest that universal selenium supplementation is inadvisable and that, where supplementation is considered, intervention should be guided by baseline screening. Animal experiments provide supporting evidence: Lei et al. [74] found that selenium deficiency promotes sodium reabsorption and elevates blood pressure via downregulation of GPX1, enhancement of oxidative stress, and NF-κB activation, and Yang et al. [75] found that selenium deficiency directly reduces the activity of selenoenzymes such as TrxR, which impairs the ability of placental and pancreatic tissues to clear lipid peroxides [11], induces oxidative stress, and thereby damages pancreatic β-cell function and exacerbates insulin resistance [76].

During pregnancy, maternal selenium requirements increase substantially, driven by multiple interrelated factors: hemodilution secondary to plasma volume expansion, enhanced renal selenium excretion, elevated oxidative stress burden, and the accelerating metabolic demands of fetal growth and development [7,17]. Serum or plasma total selenium concentration remains the most widely used biomarker of selenium status and reflects recent exposure, on the scale of weeks. Whole blood selenium indicates medium- to long-term (months) body stores, whereas selenoprotein P (SELENOP) and glutathione peroxidase 3 (GPX3) activity are regarded as more specific indicators of functional selenium status, that is, of the adequacy of selenoprotein synthesis [17,77]. In addition to SELENOP, selenoprotein S (SELENOS) is another dietary selenium transport protein. As an endoplasmic reticulum transmembrane protein, SELENOS is a core component of the endoplasmic reticulum-associated degradation (ERAD) pathway and participates in the unfolded protein response, regulation of oxidative stress, and inflammatory signaling. Unlike SELENOP, which primarily reflects medium- to long-term selenium reserves, SELENOS expression and function are more closely linked to cellular stress states and vascular homeostasis. It may therefore have independent biomarker value in stress-related pathological contexts such as hypertensive disorders of pregnancy [78].

The clinical implications of these observations depend fundamentally on baseline selenium status. Correcting established selenium deficiency—in regions where serum selenium is commonly below 70 μg/L, or in individuals with biochemically confirmed low selenium—is supported by evidence that repletion restores selenoprotein activity and may reduce oxidative stress and thyroid autoimmunity. In contrast, routine universal supplementation irrespective of baseline status is not supported: RCTs in selenium-adequate populations have consistently failed to show benefit, and intake above the saturation threshold may confer no additional benefit and could increase risk. The evidence therefore supports a targeted, status-guided approach in populations or individuals at high risk of deficiency, rather than universal screening-and-supplementation.

The impact of iodine on pregnancy outcomes is modulated substantially by thyroid compensatory capacity. This review found that isolated iodine deficiency (UIC < 150 μg/L) is not consistently associated with adverse outcomes; the true risk increase occurs only in subgroups in which iodine depletion is sufficient to disrupt thyroid homeostatic compensation. For instance, Nazarpour et al. [8] showed that preterm birth risk increased significantly only when UIC < 100 μg/L was combined with TSH ≥ 4 μIU/mL. This phenomenon is further explained by Johannesen et al. [43], who showed that serum Tg increased significantly only at UIC < 50 μg/L, while pregnant women with UIC in the 50–149 μg/L range showed no difference in thyroid function compared with the iodine-adequate group (≥150 μg/L). It should be emphasized that the WHO threshold of UIC < 150 μg/L is a population-level indicator for assessing iodine nutrition among pregnant women, rather than an individual diagnostic criterion. The findings of Johannesen et al. suggest that, in mildly to moderately iodine-deficient areas, applying this population threshold may overestimate the prevalence of functional thyroid impairment at the population level; however, this does not invalidate the threshold as a monitoring tool. Rather, it indicates that population iodine status assessment may benefit from complementary functional indicators such as serum Tg, and that individual iodine status cannot be reliably determined from a single UIC measurement. Furthermore, the U-shaped association of iodine with GDM [50,51] and the high-iodine-induced Wolff–Chaikoff effect both emphasize the clinical importance of maintaining iodine nutrition within the optimal window.

**Figure 2:**
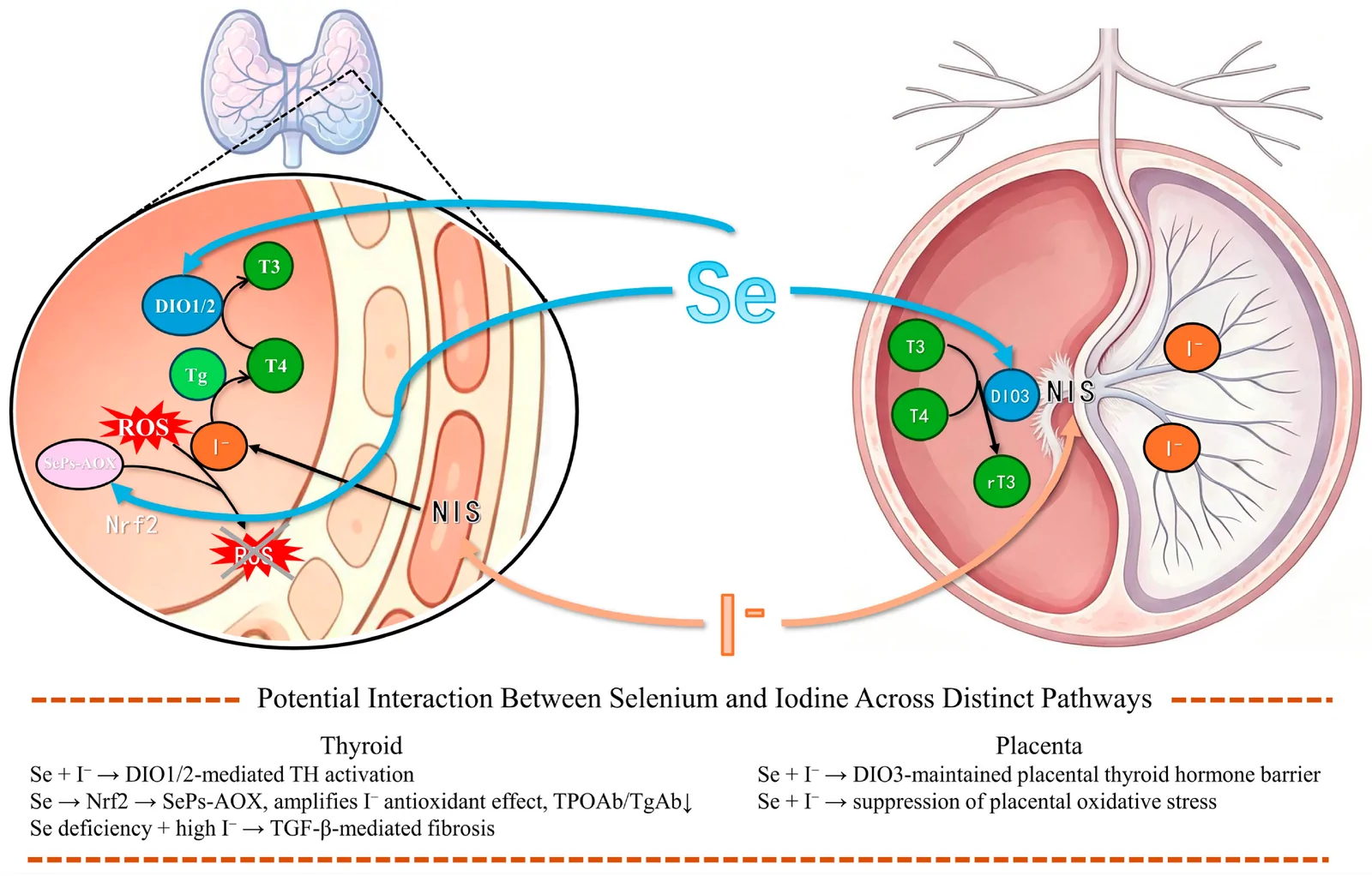
Proposed interactive pathways of selenium (Se) and iodine (I^−^) in regulating maternal thyroid function and placental homeostasis.

In placental function regulation, the sodium/iodide symporter (NIS) mediates active placental iodine uptake from maternal blood, while DIO3 forms an enzymatic barrier that limits excessive transplacental transfer of active TH [79]; selenium deficiency disrupts DIO3 expression and placental iodine homeostasis [70]. The potential interactions at these mechanistic levels have been preliminarily validated in vitro and in animal experiments, and combined supplementation was superior to single supplementation in alleviating trophoblast oxidative damage. Peng et al. [50] and Fu et al. [51] confirmed that the placenta actively concentrates iodine and that placental iodine storage correlates positively with maternal UIC and serum iodine concentrations, suggesting that NIS-mediated placental iodine transport is active during pregnancy. Wang et al. [25] found that maternal selenium levels in the second trimester were negatively correlated with placental mRNA expression of stress and inflammatory markers such as HO-1, HIF1α, and GRP78. In populations with RM, Sheikh et al. [30] observed significant downregulation of antioxidant enzyme genes, including GPX, GSR, SOD, and CAT, in placental tissues, while Ghneim et al. [29] found that placental selenium levels in patients with RM were approximately 18–29% lower than in healthy pregnant women. Figure 2 illustrates the putative interactive pathways of selenium and iodine in modulating thyroid hormone metabolism and placental function, which are the two principal axes linking maternal micronutrient status to perinatal outcomes.

These potential interactions, however, have received almost no validation at the population level. The only two population studies that concurrently measured selenium and iodine either reported association trends of selenium with thyroid hormones without clinical outcomes, or merely described the prevalence of co-deficiency. No RCT has evaluated the incremental clinical benefit of combined selenium and iodine supplementation relative to single supplementation or placebo. These studies suggest the existence of combined effects of selenium and iodine in both thyroid metabolism and placental function regulation, but whether these synergistic phenomena translate into measurable clinical benefits in humans remains to be validated by large-scale prospective cohorts and rigorously designed combined intervention studies.

Current research on selenium and iodine has several limitations. First, UIC reflects short-term iodine intake (hours to days), whereas thyroid function and fetal neurodevelopment are influenced by long-term iodine homeostasis. Although serum or whole blood selenium can reflect longer-term exposure, detection standards are not yet fully harmonized across laboratories, complicating cross-study comparisons. Second, in existing RCTs of selenium supplementation, intervention doses range from 42 μg/d to 200 μg/d, the gestational age at initiation varies widely from the first trimester to 16 weeks, and most trials do not report precise stratification of baseline selenium status, potentially masking true effects. Third, most existing studies analyze the independent effects of iodine and selenium separately; the conclusions regarding their synergistic regulation of thyroid metabolism and placental function rely solely on in vitro and animal experiments, and large-scale population cohorts and combined intervention RCT data are lacking, making it difficult to determine the strength of the synergistic effect on maternal and infant outcomes in real-world populations.

Future research should therefore: (1) collect multiple types of selenium and iodine data simultaneously in the first trimester and conduct dynamic monitoring through the second and third trimesters to investigate their trajectories throughout pregnancy and the dose–response relationship of combined exposure with composite adverse pregnancy outcomes; and (2) conduct baseline-stratified RCTs comparing the independent and interactive effects of four groups (selenium alone, iodine alone, combined supplementation, and placebo) on adverse maternal and infant outcomes.

This review also has limitations. As a scoping review, it did not conduct a systematic risk-of-bias assessment of the included literature, nor a quantitative meta-analysis; it therefore cannot provide pooled effect sizes for clinical decision-making. Additionally, only the PubMed database was searched and only English-language literature was included, which may introduce selection bias and potentially miss relevant studies published in non-English journals or other databases.

## 5. Conclusions

Physiological and metabolic changes during pregnancy simultaneously increase the requirements for selenium and iodine. Mild-to-moderate isolated or combined deficiencies of both elements are prevalent among pregnant women globally and in China. This dual nutritional imbalance, with its overlapping effects, further amplifies the risk of various adverse pregnancy outcomes and represents a significant maternal and infant public health issue requiring urgent attention. Selenium primarily exerts its effects by regulating thyroid autoimmunity and placental oxidative stress, while iodine influences thyroid hormone synthesis and the placental hormonal barrier; both elements exhibit a U-shaped dose–response relationship, with either insufficiency or excess detrimental to maternal and infant health. Mechanistically, selenium and iodine may interact at multiple levels in TH activation, antioxidant pathways, and regulation of the placental–maternal interface, and dual deficiency may compromise compensatory pathways and thus warrants particular concern; whether the combined effect exceeds the sum of individual effects remains to be tested. Current population studies, however, predominantly focus on single elements; large-scale cohort and stratified supplementation RCT evidence for combined exposure is critically insufficient, and the clinical translation of synergistic mechanisms lacks human validation. Future efforts should establish standardized approaches for the simultaneous assessment of selenium and iodine nutrition during pregnancy, conduct combined intervention trials with verification of interactive mechanisms in placental tissue, inform the development of micronutrient management strategies during the first 1000 days of life, and provide an evidence base for reducing related adverse pregnancy outcomes and developmental impairments in offspring.

## Figures and Tables

**Table 1:** Summary of interventional studies on maternal selenium nutritional status and maternal and infant outcomes.

Author (Year)	Country	Study Design	Intervention Protocol	Initiation & Duration	Main Outcomes
**Mantovani et al. (2019) [20]**	Italy	RCT	83 μg/d L-selenomethionine vs. placebo	Mid-late pregnancy to 6 months postpartum	Improved thyroid autoimmune status and reduced thyroid autoantibody levels (TgAb and TPOAb, both *p* < 0.01).
**Mesdaghinia et al. (2023) [21]**	Iran	RCT	200 μg/d selenium vs. placebo	From 16–18 weeks gestation × 12 weeks	Improved uterine artery blood flow resistance (β = −0.09, *p* = 0.04) and systemic oxidative and inflammatory status in high-risk women.
**Okunade et al. (2021) [27]**	Nigeria	RCT	200 μg/d selenium vs. placebo	During pregnancy	Reduced the risk of preterm birth in HIV-infected pregnant women (RR = 0.32, 95% CI 0.11–0.96).

**Table 2:** Summary of observational studies on maternal selenium nutritional status and maternal and infant outcomes.

Author (Year)	Country	Study Design	Exposure Assessment	Main Outcomes
**Filipowicz et al. (2022) [17]**	Poland	Cross-sectional	Third-trimester serum selenium levels	Maternal selenium status is transferred to offspring (all *p* < 0.001), but low-dose supplementation (<55 μg/d) was insufficient to correct deficiency.
**Chung et al. (2024) [19]**	South Korea	Prospective cohort	Mid-late pregnancy plasma Se < 70 vs. ≥100 μg/L	Selenium-deficient women had a higher risk of thyroid autoimmunity (TPOAb positivity, *p* = 0.031) and greater thyroid ultrasound parenchymal heterogeneity (*p* = 0.042).
**Wang et al. (2022) [25]**	China	Prospective cohort	Second-trimester maternal selenium levels	Maternal selenium levels correlated negatively with placental oxidative stress and inflammation markers (HO-1, HIF1α, GRP78; β = −0.011 to −0.005, all *p* < 0.05).
**Lewandowska et al. (2019) [22]**	Poland	Prospective cohort	First-trimester serum selenium levels	Low first-trimester selenium was a strong independent risk marker for hypertensive disorders of pregnancy (AOR = 15.34, 95% CI 2.73–86.31).
**Liu Y et al. (2023) [23]**	China	Nested case–control	Second-trimester urinary selenium levels	Low urinary selenium was associated with a higher risk of gestational diabetes mellitus (OR = 2.35, 95% CI 1.36–4.06).
**Liu P et al. (2020) [24]**	China	Prospective cohort	First-trimester serum selenium levels	No significant association between first-trimester serum selenium and gestational diabetes mellitus (OR = 1.511, 95% CI 0.815–2.803).
**Ghneim et al. (2016) [29]**	Saudi Arabia	Case–control	Plasma selenium levels during pregnancy	Systemic and placental selenium levels were significantly lower in women with recurrent miscarriage than in healthy pregnant women (all *p* < 0.001).
**Sheikh et al. (2019) [30]**	Saudi Arabia	Case–control	Plasma and placental selenium levels during pregnancy	Placental antioxidant defense capacity was reduced in women with recurrent miscarriage (gene expression downregulated, *p* < 0.001).
**Lewandowska et al. (2019) [31]**	Poland	Nested case–control	First-trimester serum selenium levels	Low first-trimester selenium (≤56.6 μg/L) was an independent risk factor for delivering small-for-gestational-age infants (OR = 3.02, *p* = 0.019).
**Demircan et al. (2024) [32]**	Denmark	Prospective cohort	Third-trimester serum total selenium levels	Higher maternal selenium was associated with a lower risk of ADHD traits (OR = 0.76, 95% CI 0.60–0.94) and ASD traits (OR = 0.85, 95% CI 0.74–0.98) in offspring.
**Polanska et al. (2016) [33]**	Poland	Prospective cohort	First-trimester plasma selenium levels	First-trimester selenium levels correlated positively with early motor (β = 0.2, *p* = 0.002) and language development (β = 0.2, *p*= 0.03) in offspring.
**Lee et al. (2021) [34]**	USA	Prospective cohort	Maternal erythrocyte selenium levels during pregnancy	In high-selenium populations, higher maternal selenium was associated with an increased risk of ASD (OR = 1.49, 95% CI 1.09–2.02) and ADHD (OR = 1.29, 95% CI 1.04–1.56) in offspring.

**Table 3:** Summary of interventional studies on maternal iodine nutritional status and maternal and infant outcomes.

Author (Year)	Country	Study Design	Intervention Protocol	Initiation & Duration	Main Outcomes
**Manousou et al. (2021) [41]**	Sweden	RCT	150 μg/d iodine vs. placebo	First trimester to late pregnancy	Alleviated thyroid burden, with limited direct effects on routine thyroid function measures.
**Gowachirapant et al. (2017) [57]**	Thailand/India	RCT	200 μg/d iodine vs. placebo	First trimester to 5 years postpartum	In mildly to moderately iodine-deficient populations, antenatal iodine supplementation did not improve preschool intelligence or executive function in offspring.

**Table 4:** Summary of observational studies on maternal iodine nutritional status and maternal and infant outcomes.

Author (Year)	Country	Study Design	Exposure Assessment	Main Outcomes
**González-Martínez et al. (2023) [42]**	Spain	Cross-sectional	Iodized salt users vs. non-users	Effects depended on autoimmune background: lower TSH in women without autoimmune thyroid disease, but higher TSH in those with the disease.
**Johannesen et al. (2025) [43]**	Faroe Islands	Cross-sectional	UIC stratification: <50, 50–149, ≥150 μg/L	TSH increased with iodine intake, whereas Tg increased only in severe iodine deficiency (UIC < 50 μg/L), suggesting current deficiency thresholds may overestimate functional impairment.
**Peng et al. (2020) [50]**	China	Cross-sectional	Maternal vs. fetal side placental iodine content	The placenta actively concentrates iodine; maternal-side iodine content exceeded fetal-side content.
**Fu et al. (2024) [51]**	China	Cross-sectional	Grouped by different iodine nutritional status	Placental iodine storage correlated positively with maternal iodine nutrition and may serve as an indicator of maternal iodine status.
**Businge et al. (2025) [44]**	DRC	Case–control	UIC levels in preeclampsia group	Low iodine exposure was associated with a higher risk of preeclampsia; UIC showed good screening efficacy (AUC = 0.920).
**Abel et al. (2020) [45]**	Norway	Prospective cohort	Dietary iodine <100 μg/d	Low iodine intake was associated with increased preeclampsia risk (AOR = 1.14, 95% CI 1.08–1.22); long-term pre-pregnancy iodine supplementation reduced the risk (AOR = 0.85).
**Wikerholmen et al. (2025) [46]**	Norway	Prospective cohort	Iodine intake <100 μg/d	Low iodine intake increased the risk of pharmacologically treated hypertension within 10 years postpartum (AHR = 1.29), even without pregnancy complications.
**Queiroz et al. (2025) [47]**	Brazil	Cross-sectional	UIC in HDP pregnant women	Iodine deficiency was prevalent in HDP; higher systolic blood pressure was associated with lower UIC, independent of thyroid function.
**Vítková et al. (2024) [48]**	Czech Republic	Prospective cohort	GDM group vs. control group UIC	In iodine-deficient areas, iodine deficiency was more prevalent in women with GDM than in healthy pregnant women.
**Liu Z et al. (2025) [49]**	China	Prospective cohort	First-trimester serum iodine concentration	In iodine-sufficient areas, higher early-pregnancy iodine exposure was associated with elevated blood glucose.
**Nazarpour et al. (2020) [8]**	Iran	Prospective cohort	UIC in hypothyroid pregnant women	Severe iodine deficiency combined with hypothyroidism increased preterm birth risk (OR = 2.5, 95% CI 1.1–10.0), whereas moderate deficiency alone did not.
**Lopes-Pereira et al. (2022) [52]**	Portugal	Retrospective cohort	Before and after iodine supplementation policy recommendation	Following nationwide promotion of supplementation, adverse outcomes such as miscarriage decreased (OR = 0.791, 95% CI 0.651–0.960).
**Zhao Y et al. (2025) [53]**	China	Cross-sectional	UIC <150 μg/L (deficiency 59.4%)	Iodine deficiency was not significantly associated with miscarriage (OR = 0.69, *p* = 0.487); the excess iodine group had a higher risk of thyroid autoimmunity.
**Huo et al. (2025) [54]**	China	Prospective cohort	Late pregnancy I:Cr <150 μg/g	Late-pregnancy iodine deficiency was associated with lower weight and length at birth and at 18 months of age.
**Snart et al. (2020) [55]**	UK	Prospective cohort	Pregnancy iodine/creatinine ratio (I:Cr)	Higher iodine status was associated with higher birth weight (+41 g) and lower SGA risk.
**Chen R et al. (2018) [56]**	China	Prospective cohort	First-trimester I:Cr < 150 μg/g	First-trimester iodine deficiency was associated with slower fetal skeletal growth in the second and third trimesters.

## Data Availability

No new data were created or analyzed in this study. Data sharing is not applicable to this article.

## Supplementary Materials

The following supporting information can be downloaded at: https://www.mdpi.com/article/10.3390/nu18213521/s1, Table S1 Preferred Reporting Items for Systematic reviews and Meta-Analyses extension for Scoping Reviews (PRISMA-ScR) Checklist.

## Abbreviations

ADHD	attention deficit hyperactivity disorder
ASD	autism spectrum disorder
CAT	catalase
DIO	iodothyronine deiodinase
GDM	gestational diabetes mellitus
GPX	glutathione peroxidase
GSR	glutathione reductase
HDP	hypertensive disorders of pregnancy
PTB	preterm birth
RM	recurrent miscarriage
ROS	reactive oxygen species
RR	risk ratio
SELENOP	selenoprotein
TH	thyroid hormone
T3	triiodothyronine
T4	thyroxine
FT4	free thyroxine
Tg	thyroglobulin
TgAb	thyroglobulin antibody
TPOAb	thyroid peroxidase antibody
TrxR	thioredoxin reductase
TSH	thyroid-stimulating hormone
UIC	urinary iodine concentration
SGA	small for gestational age
SIC	serum iodine concentration
SOD	superoxide dismutase
